# Detection of clonal hematopoiesis of indeterminate potential via genome or exome sequencing underestimates disease associations

**DOI:** 10.1172/JCI198861

**Published:** 2026-01-13

**Authors:** Robert Corty, Yash Pershad, J. Brett Heimlich, Caitlyn Vlasschaert, Leo Luo, Taralynn Mack, Kaushik Amancherla, Cassianne Robinson-Cohen, Michael Savona, Alexander G. Bick

**Affiliations:** 1 Department of Internal Medicine, Vanderbilt University Medical Center, Nashville, Tennessee, USA.; 2 Department of Internal Medicine, Queen’s University, Kingston, Ontario, Canada.; 3 Department of Radiation Oncology, Vanderbilt University Medical Center, Nashville, Tennessee, USA.; 4 University of Washington, Seattle, Washington, USA.; 5 Vanderbilt-Ingram Cancer Center, Vanderbilt University Medical Center, Nashville, Tennessee, USA.

**Keywords:** Aging, Genetics, Epidemiology, Hematopoietic stem cells

## Abstract

Epidemiology studies underestimate the strength of the association between clonal hematopoiesis and disease due to false negatives from shallow, whole-genome sequencing versus deep targeted sequencing.

**To the editor:** Clonal hematopoiesis of indeterminate potential (CHIP) occurs when greater than or equal to 4% of nucleated blood cells harbor a somatic mutation in a leukemogenic gene (1). The gold standard method to detect CHIP is deep (greater than 1,000 ×) sequencing of peripheral blood (2, 3). Researchers have detected CHIP using shallow (approximately 35 ×) sequencing of genomes or exomes in large biobanks and found associations of CHIP with numerous diseases, including hematologic malignancy, cardiovascular disease, kidney disease, and all-cause mortality (4, 5). However, the sensitivity and specificity of genome-sequencing–based CHIP detection and effects of CHIP ascertainment errors on CHIP-attributable risk are unknown. Accurate estimation of CHIP-attributable risk for disease is critical for risk stratification and disease monitoring.

To characterize performance of genome-sequencing–based CHIP detection, we performed genome and deep sequencing on identical samples from 6,336 research participants. Using empiric sensitivity and specificity of genome-sequencing–based CHIP detection, we simulated how ascertainment error influences power and precision of CHIP-disease association studies. For deep sequencing, we performed error-corrected targeted sequencing of CHIP driver gene exons with median depth after deduplication of approximately 1700 × (2). Genome sequencing was performed targeting 30 × median depth. *Mutect2* was used to call somatic mutations in CHIP driver regions. We filtered by read depth (greater than or equal to 100 for deep sequencing and greater than or equal to 15 for genome sequencing), variant allele read depth (greater than or equal to 3 for deep sequencing and greater than or equal to 2 for genome sequencing), double-strand support, and inconsistency with germline heterozygosity and variant allele fraction (VAF) greater than or equal to 2% (4).

Among 6,336 participants, we identified 629 mutations in 564 people (8.9%) by genome sequencing and 1,509 mutations in 1,255 people (19.8%) by deep sequencing. Driver-mutation frequencies were similar between genome and deep sequencing (Supplemental Figures 1 and 2; supplemental material available online with this article; https://doi.org/10.1172/JCI198861DS1). We calculated sensitivity and positive predictive value (PPV) of genome-sequencing–based CHIP calling on a per variant level using deep-sequencing–based calls as gold standard and genome-sequencing–based calls as index test. Genome-sequencing–based CHIP calling had sensitivity of 66% (417 of 629), specificity of 82% (4,950 of 6,042), and PPV of 28% (417 of 1,509) (Supplemental Figure 3). Performance metrics were highly clone-size dependent. Sensitivity was 9% for VAF 2%–5%, 32% for VAF 5%–10%, 65% for VAF 10%–20%, and 85% for VAF > 20% (Figure 1A). PPV was 0%, 43%, 66%, and 80% respectively (Figure 1B).

We performed simulations to determine how ascertainment errors influence CHIP-disease associations. For each scenario, we simulated 100,000 persons with random sex, random age (40–79 years), and age-calibrated CHIP status and VAF 1,000 times (Supplemental Methods).

First, we tested how ascertainment errors impact CHIP associations with disease prevalence. We simulated disease prevalence based on age, sex, and CHIP status for odds ratios (ORs) ranging from 1.0 to 3.0. We tested for CHIP-disease association using multivariate logistic regression. With deep-sequencing–based CHIP calls, power was 100% and OR estimation was near perfect (Figure 1, C and D). Using all genome-sequencing–based CHIP calls, the power was 5%, 25%, 46%, and 71% for a disease with CHIP-associated OR of 1.5, 2.0, 2.5, and 3.0. Power was higher when people with genome-sequencing–estimated VAF less than 10% were excluded, consistent with the high rate of false positives (Figure 1E). Using genome-sequencing-based CHIP calls, estimated ORs captured approximately 16% of true liability (Figure 1F).

Second, we tested how ascertainment errors impact CHIP associations with disease incidence. We simulated age of disease onset and age of censoring based on age, sex, and CHIP status for hazard ratios (HRs) ranging from 1.0 to 3.0. We tested for CHIP-disease association using Cox proportional hazards regression. With deep-sequencing–based CHIP calls, power was 100% and the estimated HR captured 73% of the true HR, consistent with a known downward bias in Cox regression (Supplemental Figure 4). Using genome-sequencing–based CHIP calls, the power was 8%, 28%, 53%, and 70% for a disease with CHIP-associated HR of 1.5, 2.0, 2.5, and 3.0, respectively (Supplemental Figure 4). Power increased after excluding people with genome-sequencing–estimated VAF less than 10%. Estimated HRs using genome-sequence–based CHIP calls captured approximately 9% of the true disease hazard (Supplemental Figure 4). The association between CHIP and chronic kidney disease using Cox regression in our cohort were consistent with the simulation results (genome-sequencing HR: 1.09; 95% CI: 0.94–1.26, *P* = 0.26 versus deep-sequencing HR: 1.18, 95% CI: 1.07–1.32, *P* = 0.002).

Our study has several implications. First, accurate estimation of association strength between CHIP and disease necessitates sensitive CHIP detection, which is not possible with genome-sequencing–based CHIP ascertainment. Regardless of cohort size, ascertainment errors lead to underestimation of the strength of CHIP-disease association. Second, since most well-powered CHIP epidemiology studies reanalyze genome or exome sequencing, widely cited associations between CHIP and disease risk are markedly underestimated. Given that sequencing of exomes is often deeper than genomes, exome sequencing estimates may have less bias. Studies with deep sequencing are necessary to understand the true association between CHIP and disease. Third, exclusion of genome-sequencing–based CHIP mutations with VAF less than 10% is beneficial. It is reported in studies that use genome- or exome-based CHIP detection that CHIP with VAF greater than 10% carry a higher disease risk; while expanded clone size may increase disease risk, CHIP ascertainment errors exaggerate this relationship.

Therefore, errors in CHIP ascertainment in genome and exome-based CHIP calling, lead to (a) insensitive studies, which, excluding people with observed VAF less than 10% partially remedies, and (b) underestimation of CHIP-disease association strength, which only deep sequencing can remedy.

## Funding support

This work is the result of NIH funding, in whole or in part, and is subject to the NIH Public Access Policy. Through acceptance of this federal funding, the NIH has been given a right to make the work publicly available in PubMed Central.

NIH grants DP5 OD029586, R01 AG088657, R01 AG083736, and F30 AG099331.
Arthritis National Research Foundation grant 1288083 to RC.
CTSA grants UL1TR002243.

## Supplementary Material

Supplemental data

Supporting data values

## Figures and Tables

**Figure 1:**
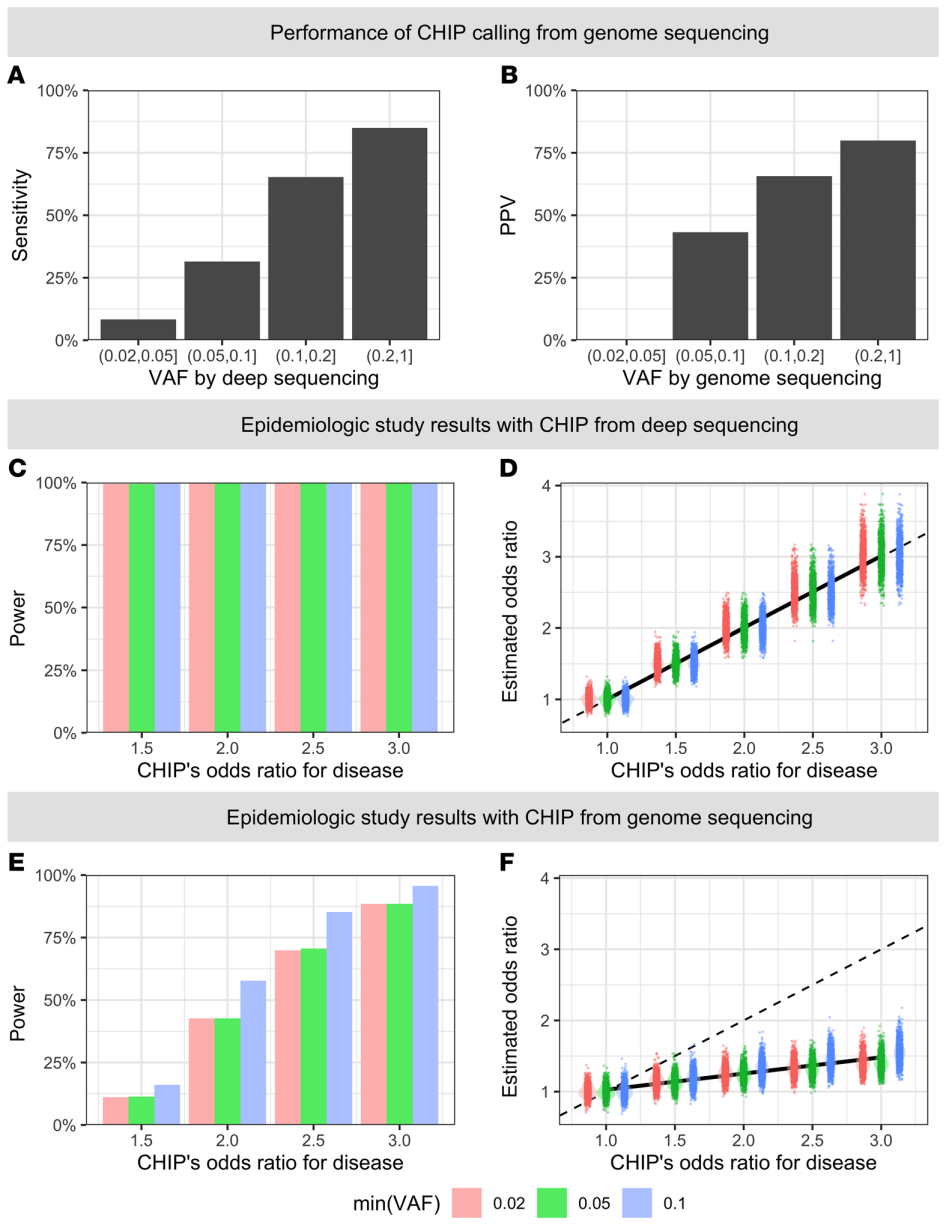
Performance characteristics of genome-sequencing–based CHIP calling and impact on epidemiologic associations. Performance of genome-sequencing–based CHIP detection compared with deep sequencing, stratified by VAF for (**A**) sensitivity and (**B**) positive predictive value. Simulated CHIP-disease associations using logistic regression across odds ratios, with minimum VAF thresholds of 0.02, 0.05, and 0.1 using deep-sequencing–based CHIP detection for (**C**) statistical power and (**D**) odds ratio estimation. Simulated CHIP-disease associations instead using genome-sequencing–based CHIP detection for (**E**) statistical power and (**F**) odds ratio estimation.

## Version 1 01/13/2026

In-Press Preview

## Version 2 03/02/2026

Electronic publication
