# Supplementary material for: Detection of clonal hematopoiesis of indeterminate potential via genome or exome sequencing underestimates disease associations
Source: J Clin Invest. 2026 Jan 13;136(5):e198861. doi: 10.1172/JCI198861 (PMC12948416; doi:10.1172/JCI198861)
Supplement: Supplemental data [file jci-136-198861-s314.pdf]

Supplementary Material

Supplemental Methods

Sex as a biologic variable:

Sex of the individuals was not considered when defining the sensitivity and positive predictive value of CHIP calls using whole-genome sequencing. In our simulation studies, individuals were assigned random sex which was used as a covariate in logistic regressions and Cox proportional hazard regressions.

Study participants:

Our study cohort was comprised of individuals who were sequenced as part of the BioVU Alliance for Genomic Discovery and sequenced using deep sequencing to detect CHIP in cohorts within BioVU as part of four prior studies. Three studies examined CHIP in specific disease states: chronic kidney disease[1], radiation therapy for solid tumors[2], and heart failure requiring transplant[3]. The fourth examined participants who donated multiple samples to study the longitudinal dynamics of CHIP[4].

Statistics:

Simulation code is provided at details are provided at github.com/bicklab/wgs_chip_is_spec_but_not_sens. In brief, plausible parameters were used to simulate study cohorts, prevalent disease analysis was conducted with a logistic model, and incident disease analysis was conducted with a Cox proportional hazards model, all in R version 4.3.2.

Simulations:

Simulations were implemented in R (version 4.3.2) using parallel processing to efficiently conduct 1,000 replicate simulations for each scenario and employed three conceptual steps: (1) generate a cohort with realistic demographic and CHIP characteristics; (2) assign disease outcomes based on specified effect size; (3) analyze the data using standard statistical models while accounting for the imperfect detection accuracy of whole-genome sequencing.

For the generation of the cohort, each simulated individual was randomly assigned sex (50% probability for each) and age uniformly distributed between 40-79 years. CHIP prevalence was modeled as age-dependent using $chip\ prevalence\ (age) = \max(0, 1 \times 10^{-6} \times (age - 20)^3)$. This polynomial equation was empirically calibrated to match observed rates: ~1% at age 40, ~10% at age 60, ~20% at age 75, and ~30% at age 85. For individuals with CHIP, variant allele fraction (VAF) was drawn from a beta distribution with $\alpha = 1$ and $\beta = 20 - 18 \times \frac{age}{100}$, bounded between 2-40%. This distribution generates realistic VAF distributions that skew toward smaller clones overall and generates a risk of larger clones that increases with age.

We modeled whole-genome sequencing's detection probability using empirically observed values from our 6,336-person comparison study. For individuals with true CHIP, detection probability varied by VAF: 9% for VAF 2-5%, 32% for VAF 5-10%, 67% for VAF 10-20%, and 86% for VAF >20%. Moreover, for CHIP mutations "detected" by whole-genome sequencing in our simulations, we modeled observed VAF as a function of true VAF to adjust for sampling variability in VAF due to the low depth-of-coverage of genome sequencing. False positive calls occurred in individuals without true CHIP with probability 6%. False positive VAF values were randomly sampled from 536 empirically observed false positives.

For prevalent disease, binary disease status was generated using logistic regression: $logit(P(disease)) = -3 + 0.03 \times age + 0.4 \times sex + log(OR) \times CHIP,$ where OR varied from 1.0 to 3.0 across simulations.

For incident disease, we used a proportional hazards framework where $individual\ hazard = \lambda_0 \times exp(0.03 \times age + 0.4 \times sex + log(HR) \times CHIP + \varepsilon)$, with $\lambda_0 = 0.01$, HR varying from 1.0 to 3.0, and $\varepsilon \sim N(0,1)$. Survival times were drawn from exponential distributions with individual-specific hazards. Censoring times were exponentially distributed with rate$0.005 + 0.002 \times (age - 40)$ $0.005 + 0.002 \times (age - 40)$, creating realistic follow-up where older individuals experienced earlier censoring.

Each replicate was analyzed using different CHIP detection strategies: (1) true CHIP status, (2) whole-genome sequencing-based calls with VAF ≥2%, (3) VAF ≥5%, and (4) VAF ≥10%. Prevalent disease analyses used logistic regression adjusting for age and sex. Incident disease analyses used Cox proportional hazards regression adjusting for age and sex. From each model, we extracted the CHIP effect estimate and p-value.

Across 1,000 replicates per true effect size, we calculated power (proportion with p<0.01) and characterized effect size estimate distributions. This quantified both the loss of power and the systematic underestimation introduced by imperfect whole-genome sequencing detection.

Association of CHIP with chronic kidney disease among study participants:

To test the underestimation of CHIP-disease associations identified in our simulation using real-world data, we tested the association between CHIP and incident chronic kidney disease (CKD) in our cohort of doubly sequenced individuals using Cox proportional hazards regression. CKD was defined by PhecodeX GU_582. Prevalent CKD was excluded for incident analysis. Covariates adjusted were age, age^2, and ever smoker (yes/no). We compared the hazard ratio and p-value of CHIP ascertained from genome sequencing calls and deep sequencing calls.

Study approval:

Vanderbilt University Medical Center's Institutional Review Board oversees BioVU and approved this project (IRB #201783).

Data availability:

Clonal hematopoiesis of indeterminate potential sequencing calls are available through controlled access to qualified researchers at https://zenodo.org/records/18023763. Due to Vanderbilt BioVU cohort restrictions, access will require a data use agreement with Vanderbilt University Medical Center, which can be facilitated by the corresponding author.

Code availability:

Analysis code and simulations are available at: github.com/bicklab/wgs_chip_is_spec_but_not_sens.

Supplemental Figures

Supplemental Figure 1:

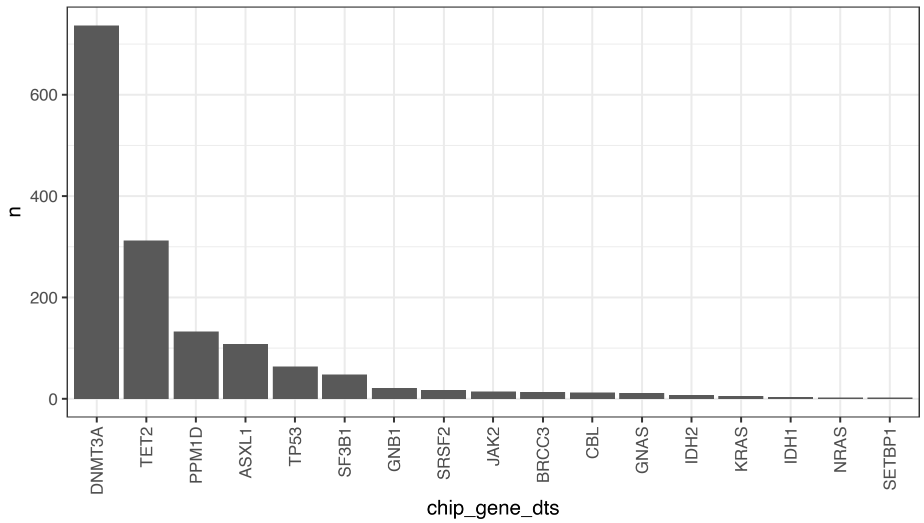

Supplemental Figure 1. Number of CHIP driver mutations found in each gene by deep, targeted sequencing

Supplemental Figure 2:

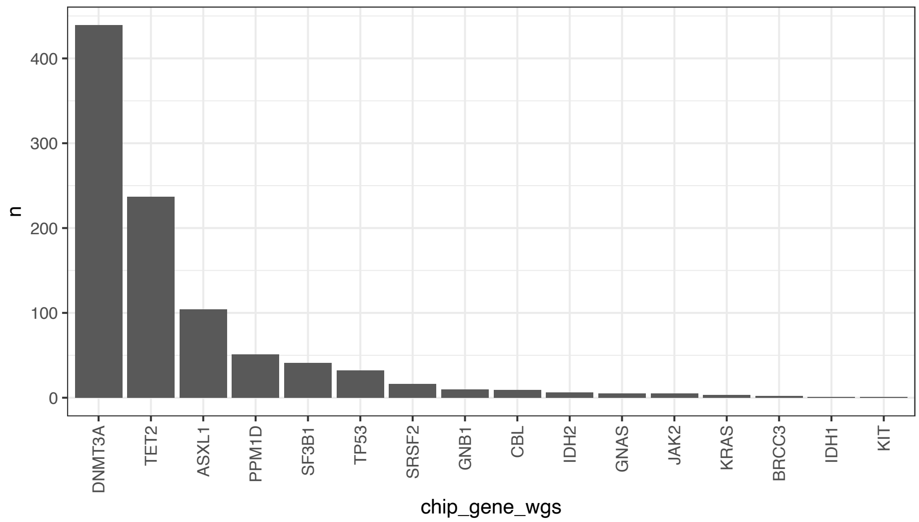

Supplemental Figure 2. Number of CHIP driver mutations found in each gene by whole genome sequencing (WGS).

Supplemental Figure 3:

		CHIP by genome sequencing (index test)		
		Positive	negative	
CHIP by deep sequencing (gold standard)	positive	True positives **417**	False negatives **212**	Sensitivity = **66.3%**
	negative	False positives **1,092**	True negatives **4,950**	Specificity = **81.9%**
		PPV = **27.6%**	NPV = **95.9%**	

Supplemental Figure 3. Confusion matrix of CHIP ascertainment by genome sequencing and deep sequencing among 6,336 doubly sequenced participants. The true negatives represent the participants who was correctly determined to have no CHIP mutations by genome-sequencing.

Supplemental Figure 4:

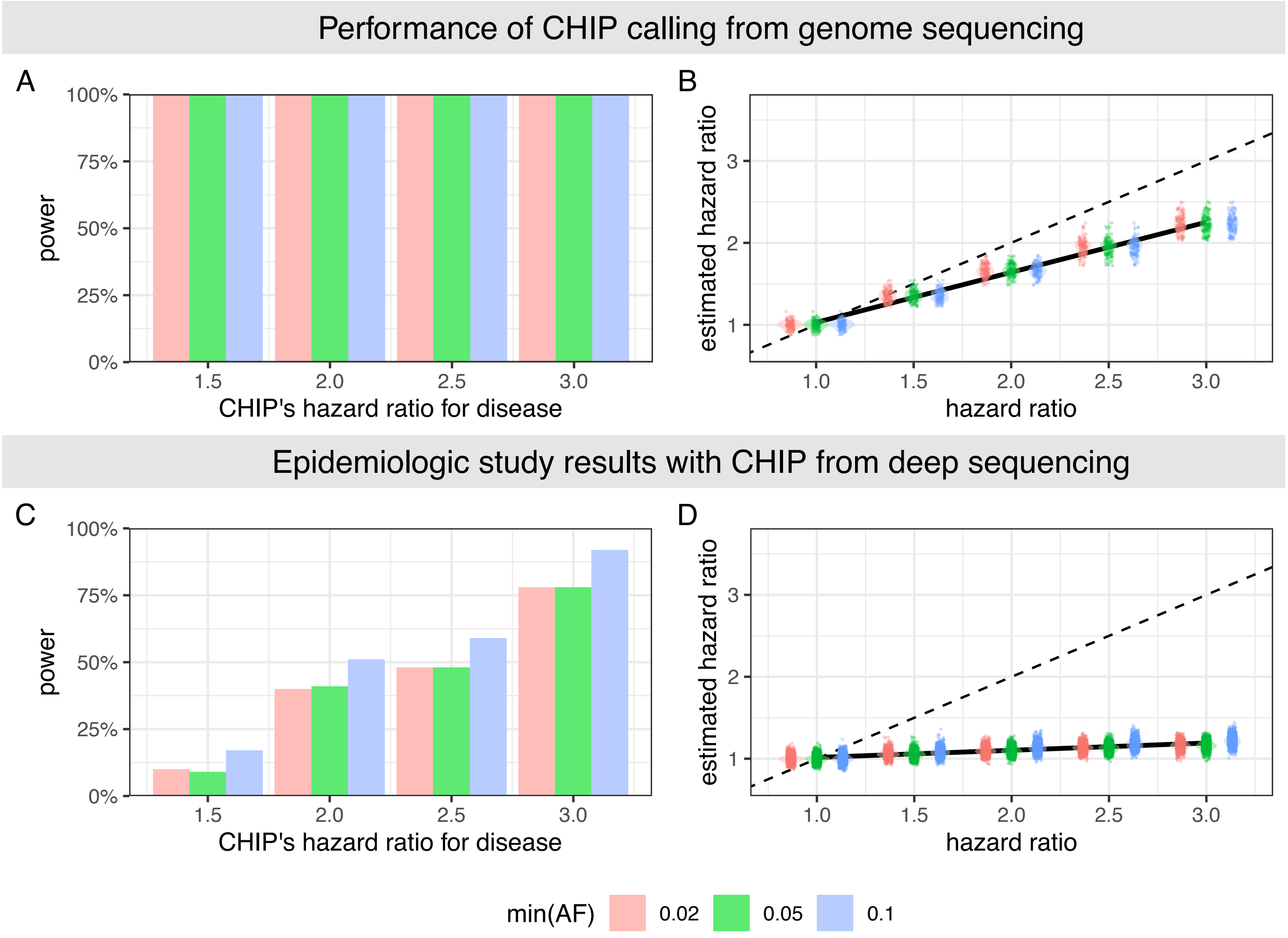

Supplemental Figure 4. In simulations, incident testing models with access to true CHIP status have 100% power to detect CHIP-disease association and much more precise estimation than models using WGS-based CHIP status. Simulated CHIP-disease associations using Cox proportional hazards regression across hazard ratios, with minimum VAF thresholds of 0.02, 0.05, and 0.1 using deep-sequencing-based CHIP detection for (A) statistical power and (B) hazard ratio estimation compared to true hazard ratio. As above, simulated CHIP-disease associations using Cox proportional hazards regression, but instead using genome-sequencing-based CHIP detection for (C) statistical power and (D) bias in hazard ratio estimation compared to true hazard ratio. As expected, survival analysis is slightly biased toward the null even with perfect CHIP ascertainment, due to the known random noise injected into the simulation studies, thereby explaining the deviation from y=x.

Funding and Acknowledgments:

Vanderbilt University Medical Center's BioVU projects are supported by numerous sources: institutional funding, private agencies, and federal grants. These include NIH funded Shared Instrumentation Grants S10OD017985, S10RR025141, and S10OD025092; and CTSA grants UL1TR002243, UL1TR000445, and UL1RR024975. Genomic data are also supported by investigator-led projects that include U01HG004798, R01NS032830, RC2GM092618, P50GM115305, U01HG006378, U19HL065962, R01HD074711.

The sequencing of 250,000 WGS individuals from BioVU®, including the 10,310 described here, has been funded by the Alliance for Genomic Discovery consisting of NashBio, Illumina and industry partners Amgen, AbbVie, AstraZeneca, Bayer, BMS, GSK, Merck, and Novo. DNA sequencing was performed at deCODE genetics using Illumina sequencing technology.

Author contributions:

RWC and YP are co–first authors. The authorship order reflects that the study was initiated by RWC, who was joined by YP in leading the project. RWC, YP, and AGB conceived the project. RWC performed statistical analyses and simulations with YP's input. CV performed CHIP calling. LYL, YP, TMM, KA, and CRC provided data from their respective studies with deep-targeted sequencing. RWC and YP drafted the initial manuscript. MRS made key conceptual contributions and critically revised the manuscript. All authors read and approved the final manuscript.

Supplemental References

1. Vlasschaert, C. *et al.* Clonal hematopoiesis of indeterminate potential is associated with acute kidney injury. *Nat. Med.* **30**, 810–817 (2024).
2. Crants, S. A. *et al.* Risk of clonal hematopoiesis of indeterminate potential after cancer radiation therapy. *medRxiv* 2024.09.27.24314321 (2024) doi:10.1101/2024.09.27.24314321.
3. Mack, T. *et al.* Germline genetics, disease, and exposure to medication influence longitudinal dynamics of clonal hematopoiesis. *Haematologica* (2024) doi:10.3324/haematol.2024.286513.
4. Amancherla, K. *et al.* Clonal hematopoiesis of indeterminate potential and outcomes after heart transplantation: A multicenter study. *Am. J. Transplant* **23**, 1256–1263 (2023).
